# Supplementary material for: Osteocyte-Derived CaMKK2 Regulates Osteoclasts and Bone Mass in a Sex-Dependent Manner through Secreted Calpastatin
Source: Int J Mol Sci. 2023 Mar 1;24(5):4718. doi: 10.3390/ijms24054718 (PMC10003151; doi:10.3390/ijms24054718)

Supplementary Figure S1: Conditional deletion 25 of CaMKK2 from osteocytes does not impact cortical bone geometry in female or male mice. (**A**) Representative crosssections of the femoral midshaft of female and male control and *Camkk2*$^{\Delta OCY}$ mice. Cortical bone parameters for female and male Control and *Camkk2*$^{\Delta OCY}$ femurs including (**Ai, Bi**) cortical bone area, (**Aii, Bii**) cross-sectional thickness and (**Aiii, Biii**) polar moment of inertia. Error bars represent SD.

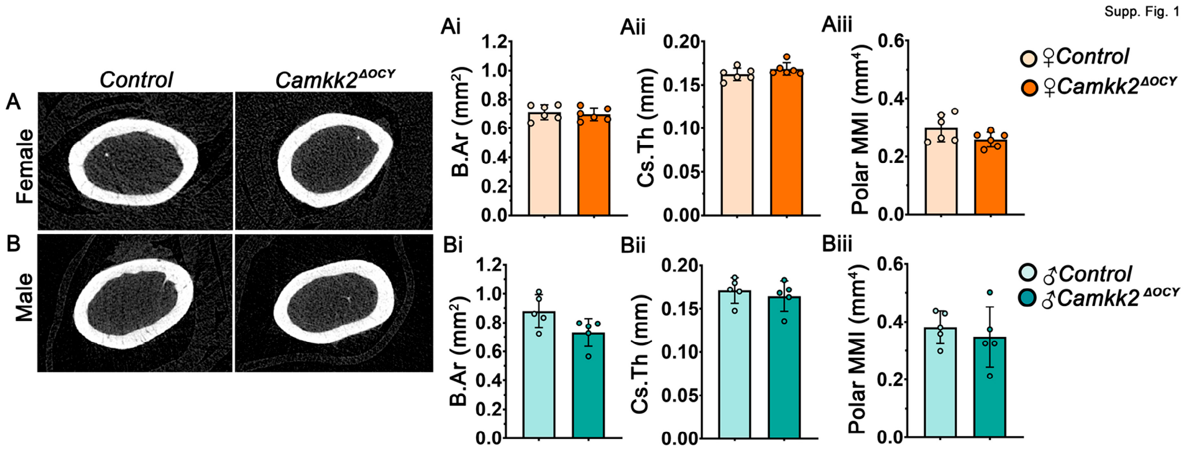

Supplementary Figure S2: **Expression of classical bone remodeling factors remain unaltered in CaMKK2-deficient osteocytes.** (**A**,**B**) Expression of *Rankl* and *Opg* mRNA, as well as the Rankl/Opg ratio in osteocytes isolated from individual female and male control and *Camkk2*$^{\Delta OCY}$ mice (n=10-12 mice/group). *Rankl* and *Opg* mRNA levels were normalized to those of *β-Actin*.

Supp. Fig. 2

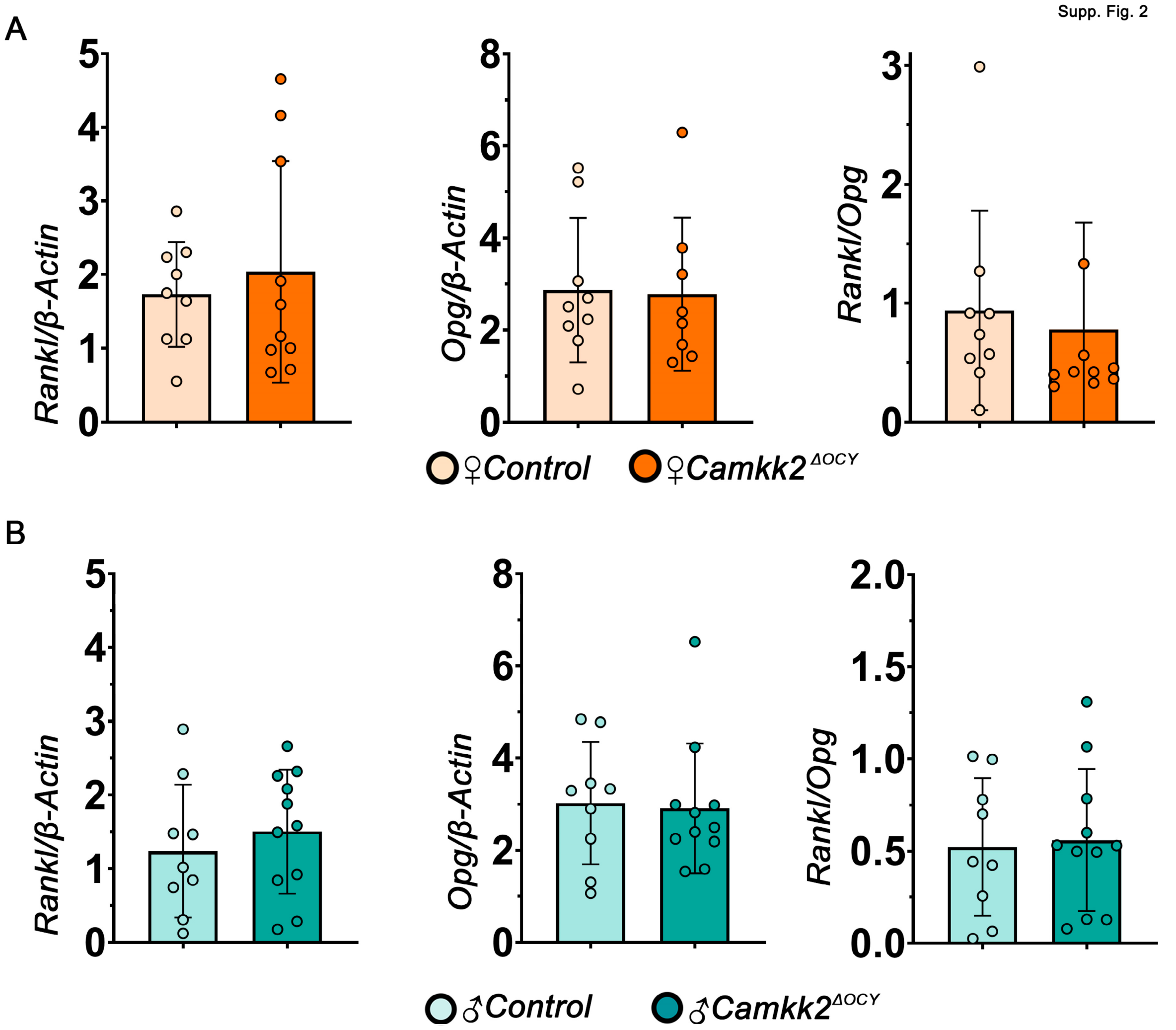

Supplementary Figure S3: Treatment with non-cell permeable recombinant calpastatin results in a dose-dependent inhibition of OCs in a sex-divergent manner. (**A**) Representative images (magnification – 40X) showing hydroxyapatite resorption pits generated by female and male WT OCs that were treated with 0, 0.1 μM, 0.5 μM, 1.0 μM and 5.0 μM NCP human calpastatin domain I. BM-derived monocytes were treated with the indicated dose of recombinant calpastatin in OC media containing RANKL and M-CSF1 for 6 days. (**B**, **E**) total number of pits measured; (**C**, **F**) percent area resorbed; and (**D**, **G**) number of TRAP-positive osteoclasts with ≥ 3 nuclei, in male and female cohorts, respectively. Error bars represent SD. * p<0.05, ** p<0.01, *** p<0.001, **** p<0.0001.

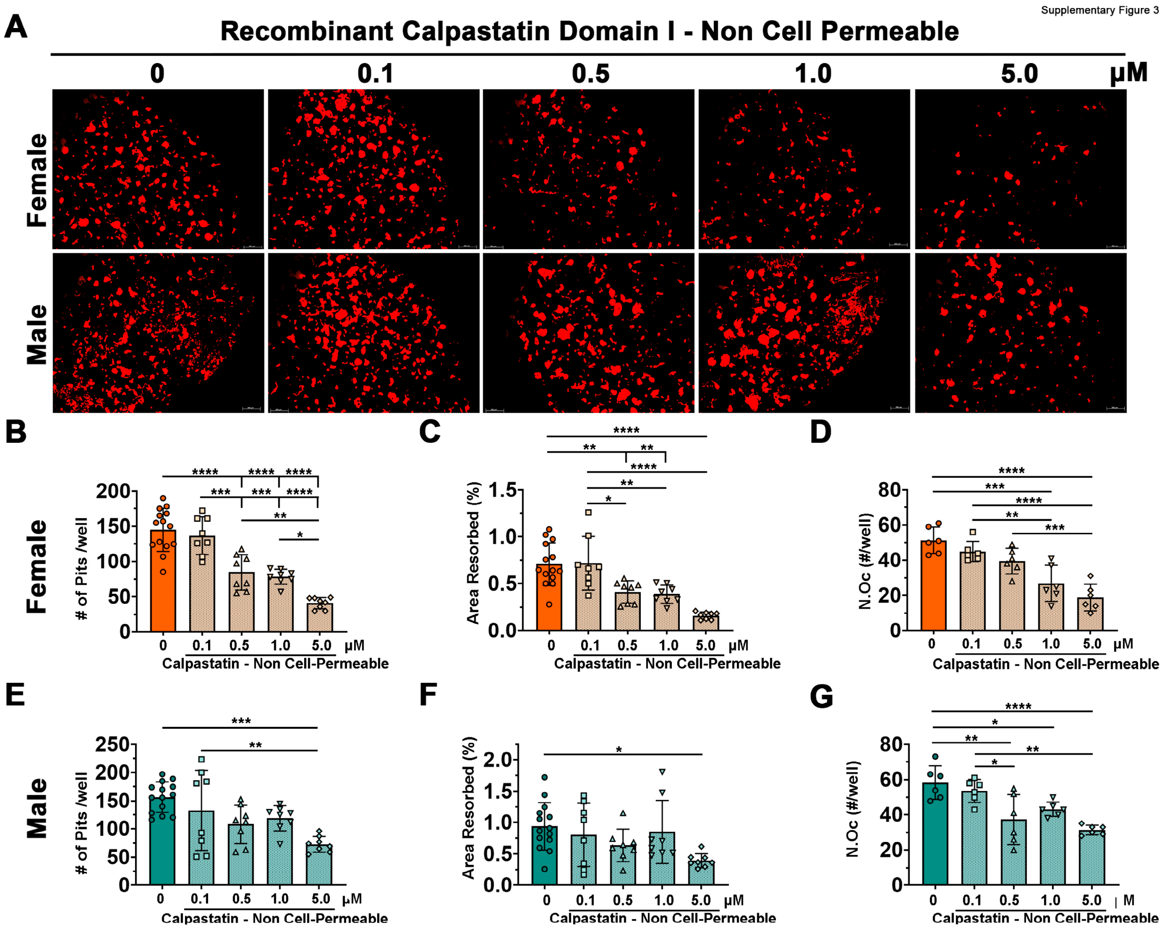

Supplementary Figure S4: Conditioned media from CaMKK2-deficient osteocytes does not affect osteoclast precursor migration or intracellular levels of Cast in OCs. (**A**-**B**) Representative phase contrast images (magnification 100X) of WT BM-derived OC precursors at (**A**) 0 hours (immediately after scratch) and (**B**) 18 hours post-scratch. (**C**) Percent of area within the scratch generated gap that was covered by OC precursors at 18 hours post-scratch as calculated using ImageJ software. (**D**) Representative i 50 mmunoblots showing levels of intracellular talin (calpain substrate), calpastatin, phosphorylated PKA-C and total PKA-C (upstream activator of calpains) relative to b-Actin in WT OCs, following treatment with female control osteocyte CM or *Camkk2*$^{\Delta OCY}$ osteocyte CM depleted of calpastatin or treated with control IgG.

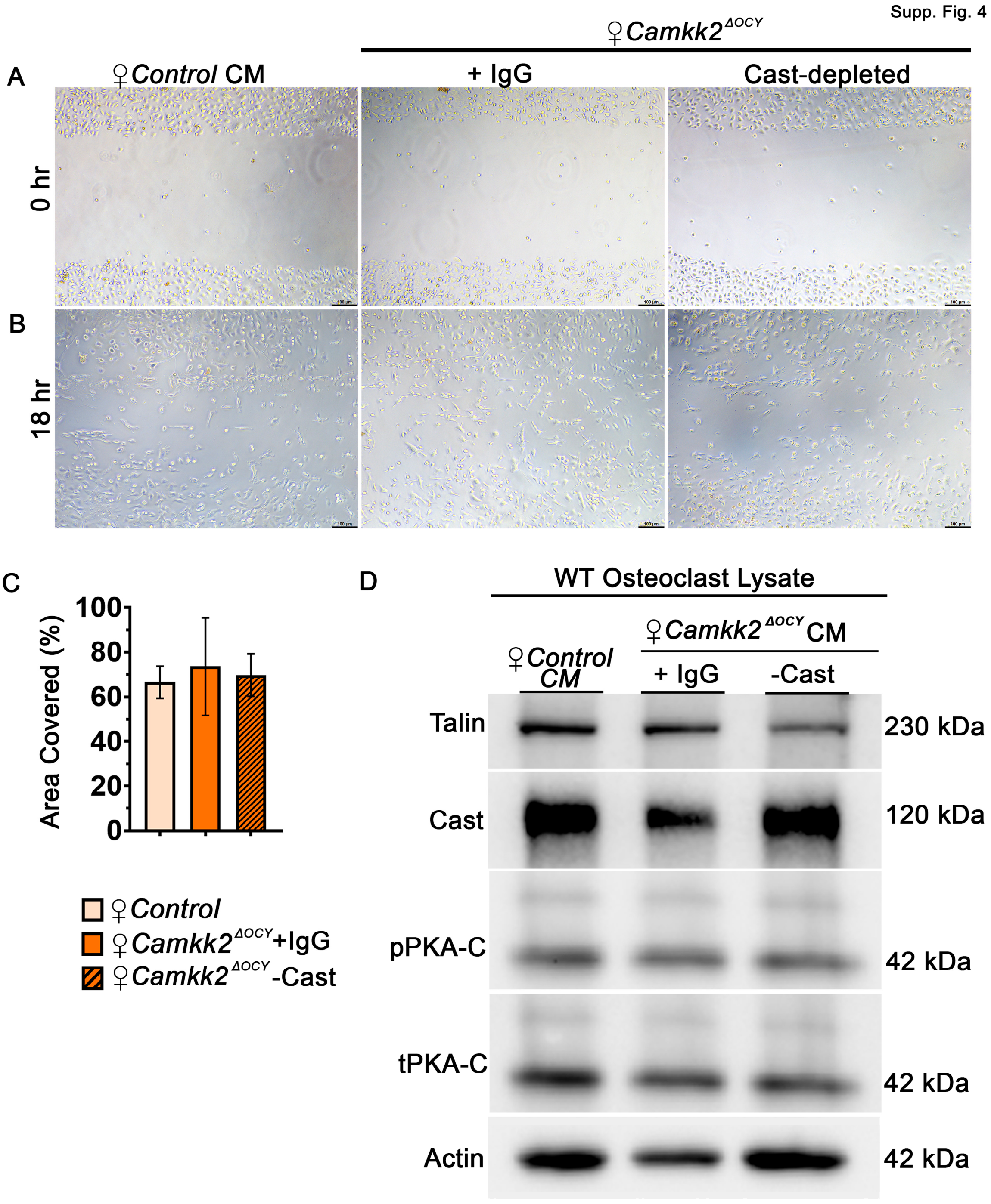

Supplement: Supplementary file 1 [file ijms-24-04718-s001.zip › ijms-2214061-supplementary.pdf]
